# Can Perceptuo-Motor Skills Assessment Outcomes in Young Table Tennis Players (7–11 years) Predict Future Competition Participation and Performance? An Observational Prospective Study

**DOI:** 10.1371/journal.pone.0149037

**Published:** 2016-02-10

**Authors:** Irene R. Faber, Marije T. Elferink-Gemser, Niels R. Faber, Frits G. J. Oosterveld, Maria W. G. Nijhuis-Van der Sanden

**Affiliations:** 1 Faculty of Physical Activity and Health, Saxion University of Applied Sciences, Enschede, The Netherlands; 2 IQ healthcare, Radboud Institute for Health Sciences, Radboud university medical center, Nijmegen, The Netherlands; 3 International Table Tennis Federation, Lausanne, Switzerland; 4 Institute for Studies in Sports and Exercise, HAN University of Applied Sciences, Nijmegen, The Netherlands; 5 Centre for Human Movement Sciences, University Medical Centre Groningen, University of Groningen, Groningen, The Netherlands; 6 International Business School, Hanze University of Applied Sciences, Groningen, The Netherlands; 7 Nijmegen School of Management, Radboud University Nijmegen, Nijmegen, The Netherlands; Research Center for Sports Sciences, Health and Human Development (CIDESD), University of Trás-os-Montes e Alto Douro, Vila Real, Portugal, PORTUGAL

## Abstract

Forecasting future performance in youth table tennis players based on current performance is complex due to, among other things, differences between youth players in growth, development, maturity, context and table tennis experience. Talent development programmes might benefit from an assessment of underlying perceptuo-motor skills for table tennis, which is hypothesized to determine the players’ potential concerning the perceptuo-motor domain. The Dutch perceptuo-motor skills assessment intends to measure the perceptuo-motor potential for table tennis in youth players by assessing the underlying skills crucial for developing technical and tactical qualities. Untrained perceptuo-motor tasks are used as these are suggested to represent a player’s future potential better than specific sport skills themselves as the latter depend on exposure to the sport itself. This study evaluated the value of the perceptuo-motor skills assessment for a talent developmental programme by evaluating its predictive validity for competition participation and performance in 48 young table tennis players (7–11 years). Players were tested on their perceptuo-motor skills once during a regional talent day, and the subsequent competition results were recorded half-yearly over a period of 2.5 years. Logistic regression analysis showed that test scores did not predict future competition participation (*p* >0.05). Yet, the Generalized Estimating Equations analysis, including the test items ‘aiming at target’, ‘throwing a ball’, and ‘eye-hand coordination’ in the best fitting model, revealed that the outcomes of the perceptuo-motor skills assessment were significant predictors for future competition results (R^2^ = 51%). Since the test age influences the perceptuo-motor skills assessment’s outcome, another multivariable model was proposed including test age as a covariate (R^2^ = 53%). This evaluation demonstrates promising prospects for the perceptuo-motor skills assessment to be included in a talent development programme. Future studies are needed to clarify the predictive value in a larger sample of youth competition players over a longer period in time.

## Introduction

Assessing the potential of young table tennis players to become elite players is extremely difficult since long-term success is less predictable because of (among other factors) the multidimensionality of the performance characteristics in elite sports and the influence of personal development and learning curves [1–3]. Nevertheless, criteria based on the maximum potential of a player are expected to be essential for the selection and monitoring of young players of successful talent development programmes to increase the effectiveness of personal coaching and increased training facilities [4–7]. At this moment, ranking position and scout’s observations often form the basis of selection criteria. Ranking position at a young age (<14 years) and junior age (<18 years / <21 years), however, has proven to be a poor indicator of a player’s potential, as it is not likely to predict future success in the long term, i.e. adult success [8–10]. Moreover, since table tennis performance itself is influenced by individual differences in growth, maturation, development and learning curves, training experiences, competition participation and environmental factors, scouts experience difficulties in finding youth players with the highest potential for elite table tennis and in explaining this explicitly [1,2]. Although measuring potential in a developing child is challenging, innovative solutions to find ‘diamonds in the rough’ might improve the success rate of talent development programmes in table tennis.

Table tennis is regarded as one of the fastest sports in terms of game speed [11]. Players aiming to excel need to develop excellent perceptuo-motor and anticipatory skills to be able to make quick and responsive adaptations to continuously changing conditions [11–15]. Moreover, they must learn to master outstanding tactical skills and withstand the physical demands [16–18]. Inseparable from this, mental aspects, such as concentration and mental toughness, need to be optimised during the extensive development programme to reach an elite level. Not to mention, volition, self-regulation, and social skills are crucial factors for persevering throughout this training process for many years [17,19–22].

Yet, although talent development in table tennis is acknowledged as a multi-dimensional process, table tennis at elite level appeals significantly to a player’s perceptuo-motor skills [23–26]. These skills are considered fundamental in developing outstanding technical skills specific to table tennis (i.e. strokes under varying circumstances) [27,28]. The optimal automation of technical skills also entails better possibilities for a player to execute tactical strategies (i.e. using an adequate solution to handle the specific demands in a situation) [29]. The complex technical skills of table tennis are learned best at the age of approximately 5 years till the pubertal growth spurt (12–14 years) [30], using the most sensitive period for learning perceptuo-motor skills [23,31,32]. Consequently, an assessment of perceptuo-motor skills as a part of a talent development programme for measuring the potential of young table tennis players seems sensible [7,23,33].

From this perspective, the Netherlands Table Tennis Association proposed a perceptuo-motor skills assessment [34]. This assessment intends to measure the potential of a young player (6–12 years) with regard to the perceptuo-motor domain, by assessing underlying perceptuo-motor skills for table tennis without using already trained authentic table tennis tasks [35,36]. Assessing these underlying perceptuo-motor skills is suggested to represent a player’s future potential better than specific sport skills themselves, which depend on exposure to the sport itself, table tennis in this case [5–7,27,35,37]. At this moment, the reproducibility of all test items is confirmed and the internal consistency and validity have good prospects [35,36]. Still, an evaluation of the perceptuo-motor skills assessment concerning its predictive validity is essential for talent development purposes [5,6]. The predictive validity should include both future competition participation and future competition performance for those who started playing official competitions. As a result, this study focuses on the following research questions:

1. Can the outcomes of the perceptuo-motor skills assessment predict future competition *participation* in young table tennis players?
2. Can the outcomes of the perceptuo-motor skills assessment predict future competition *performance* in young table tennis players?

It is hypothesized that the perceptuo-motor skills assessment outcomes predict both the possibility that children will start competition participation and the competition performance within the official competition. We set out to test the perceptuo-motor potential to become excellent table tennis players by using untrained perceptuo-motor tasks to avoid the influence of differences in table tennis experiences. Players who have better perceptuo-motor skills are suggested to be more motivated to start participation in competition and perform better during competition. Nevertheless, as other factors such as the influence of parents, the presence of local training facilities and coaches, and the availability and level of team-members are also considered to have a high impact on the table tennis performance level and the decision to participate, it is not known to what extent the perceptuo-motor skills outcomes can predict either competition participation and competition performance [38].

## Material and Methods

### Ethics Statement

This study and its informed consent procedure were approved by the ethical committee of the Medical Spectrum Twente (Medical School Twente, Institute for Applied Sciences, Enschede, the Netherlands; MTC/11069.oos 18-2-2011) in full compliance with the declaration of Helsinki. Written parental informed consent and players’ consent were obtained prior to the testing. Furthermore, both the children and their parents have given written informed consent, as outlined in the PLOS consent form, to publication of the picture (S1 File).

### Study design

An observational prospective design was used to evaluate the predictive validity of a perceptuo-motor skills assessment in young table tennis players, aged 7–11, concerning competition participation and performance outcomes. After the perceptuo-motor skills assessment, players’ competition participation and performance were monitored during five consecutive competition periods of six months.

### Players

Young table tennis players were recruited on the regional talent day of the eastern department of the Netherlands Table Tennis Association in 2011 and 2012. The eastern department is one of eight regional competition departments connected to the Netherlands Table Tennis Association. Table tennis club members of the youngest age category (≤ 11 years) present during the regional talent day were selected and registered for these events by the trainers or coaches of their local clubs. The trainers and coaches were instructed to invite the youth members of their table tennis club with the highest potential for regional and/or national elite table tennis regarding both physical and mental aspects. These players needed to be under the age of 11. The eastern department’s total population of young players was estimated to be between 100 and 120 players per year. Players with injuries were excluded from the study.

### Perceptuo-motor skills assessment

The perceptuo-motor skills assessment of the Netherlands Table Tennis Association consists of eight test items [35,36]. The standardization of the test items is captured in protocols, which includes a detailed description of materials, set-up, assignment, demonstration, training phase, testing phase and registering test scores (S1 File) [27,34–36]. ‘Sprint’ included a pyramid-shape circuit in which players need to gather and return five table tennis balls one by one as fast as possible from five different baskets starting at the basis of the pyramid-shaped circuit. Time was measured in seconds and the best of two attempts was used as the final score. For ‘agility’, players needed to get through a circuit, including climbing over a gymnastics’ cabinet (five times) and under and over a low hurdle (four times), as fast as possible. Players had one attempt in which time was measured in seconds. At ‘vertical jump’ players were instructed to stand next to a wall and jump and touch the wall with their fingertips as high as possible. The difference between the jumping height and standing height with one arm up along the wall was measured in centimetres. The best of three attempts was used as final score. ‘Speed while dribbling’ used a zigzag circuit in which the players needed to move sideways as fast as possible while dribbling with a basketball using one hand. Players had one attempt in which time was measured in seconds. At ‘aiming at target’ players needed to hit a round target (Ø 60 cm) on the floor at 2.5 meter distance with a table tennis ball using a standard bat with their preferred hand. Forehand and backhand had to be used alternately during the attempts. A hit in the target’ centre (Ø 0.20 m) or the outer ring yielded 6 and 4 points, respectively. The total score of ten attempts was registered as the final score. ‘Balls skills’ also required hitting a round target on the floor (Ø 75 cm), but now players needed to throw a table tennis ball with their preferred hand via a vertical table tennis table from two different positions (1 and 2 meter distance away from the target). Each player had a total of twenty attempts. A hit in the centre (Ø 0.335 m) or the outer ring of the target yielded 2 and 1 points, respectively. The total score of the twenty attempts was registered as the final score. At ‘throwing a ball’, the players threw a table tennis ball as far away as possible with their preferred hand. The distance from the starting-point at the marked line to the point of the ball’s first bounce was measured in meters. The best of three attempts was used as final score. In the ‘eye-hand coordination’ test players were instructed to throw a ball at a vertical table tennis table at 1 meter distance with one hand and to catch the ball correctly with the other hand as frequently as possible in 30 seconds. The number of correct catches was scored. The complete test protocol of the perceptuo-motor skills assessment is available (S1 File). To obtain a total score for the perceptuo-motor skills assessment, raw test scores were first converted into percentile scores per test item in coherence with the previous study. The total score is computed by summing up the percentile scores of all eight test items (range 0–800 points) [35].

An initial evaluation of the perceptuo-motor skills assessment demonstrated fair to good reproducibility with regard to the level of test items, except for two test items: ‘aiming at target’ and ‘ball skills’ [35]. The internal consistency of all test items was satisfactory, and the principal component analyses revealed two underlying dimensions; ‘ball control’ and ‘gross motor function’. On the first factor ‘ball control’, high loadings (>0.65) were found for ‘speed while dribbling’, ‘aiming at target’, ‘ball skills’, ‘throwing a ball’ and ‘eye-hand coordination’. On the second factor ‘gross motor function’, high loadings (>0.65) were found for ‘sprint’, ‘agility’, and ‘vertical jump’ [35]. As expected, there were moderate but significant relationships between the perceptuo-motor skills assessment’s total score and the national ranking for boys and girls (6–10 years) at the moment of testing [35]. A revision of the test items ‘aiming at target’ and ‘ball skills’ ensured reproducible test scores, which also discriminated between high and low performers in young table tennis players [36]. Consequently, both revised test items replaced the original test items in the perceptuo-motor skills assessment.

Since the perceptuo-motor skills assessment included two revised test items, the reproducibility of the total score was evaluated as a part of this study using a test-retest design (n = 53) with the internal consistency being determined on the basis of the results of the initial test (n = 53). The intraclass correlation coefficient (ICC; two-way random model (type consistency; single measurement outcome) of the total score (0.91; *p* <0.001) and the lower boundaries of the 95% confidence interval (0.90) met the criteria of >0.81 (*p* <0.05) for reliability [39]. The smallest detectable difference (SDD), and coefficient of variation (CV), used as agreement parameters [40], valued 98 points and 7%, respectively. Cronbach’s alpha was calculated at 0.82 including all test items, which meets the criteria of 0.8 for good internal consistency [41].

All players were assessed under similar conditions at a local training centre during a regional talent day. Before starting the assessments, all participants did a warming-up as a part of the event. The testers were students of physiotherapy or table tennis trainers trained to the same degree with regard to using the test protocols; they were familiarized with the test protocol. Additionally, instructions and feedback were given during a training session by an expert-trainer of the Netherlands Table Tennis Association. The participants’ characteristics of height, weight and current training hours per week, as well as the control variables of sex, age, and training experience in months were extracted from the register forms.

### Competition participation and performance

In the Netherlands, youth players can participate in the official competition of the Netherlands Table Tennis Association. This is a team competition and uses a hierarchical structure including both the national leagues and the regional leagues of eight departments. The highest national and regional leagues consist of only one group of teams. The other leagues contain more groups. In one calendar year, two competition periods are included, the autumn and the spring period, with 10 team matches each. Teams participating in the competitions consist of at least three players. Each team match contains nine individual matches in which three team members play against three players of the opponent one by one (i.e. three individual matches per player). Furthermore, two members of each team compete at the double-match. Each match won counts for one point, so a maximum score of 10 points can be obtained by a team per team match. The team with the most point in a competition period becomes champion of its league’s group and will be promoted to the subsequent higher league for the next competition period. Champions of the highest regional league will promote to the lowest national league in this case.

Competition participation and competition performance were provided by the Netherlands Table Tennis Association. Competition participation was considered nominal data (yes/no). A ‘yes’ was recorded when a player participated in an official competition of the Netherlands Table Tennis Association in at least one of the five consecutive competition periods of 6 months each after the regional talent day. Otherwise a ‘no’ was recorded.

The competition scores (points) of the five consecutive competition periods after the perceptuo-motor skills assessment were used as competition performance outcome. These competition scores per period are composite scores based on a player’s competition level and the percentage of matches he or she won during that period [42]. The calculation of the period’s competition score is based on the official Netherlands Table Tennis Association’s national and regional competitions and can be converted to international standards. All leagues of the regional and national competitions for both youth and adult players and male and female players are compared in this one system taking strength differences between the existing leagues of the official competition of the Netherlands Table Tennis Association into account. As such, a player’s table tennis performance is ranked within a certain competition period compared to all players competing in different leagues.

### Statistical analysis

IBM SPSS Statistics 21 (IBM Corp., Armonk, New York, United States of America) was used for the statistical analyses. The normality of data was evaluated by comparing (1) means and medians of the test items and (2) standard deviation and ranges. Sample characteristics and descriptive statistics of the raw test item scores and the total score, including means, standard deviations, and ranges, are presented for the total group and split up for non-competition and competition players. The influences of sex and age on the total score of the perceptuo-motor skills assessment were tested to reveal the necessity to include them as covariates using a univariable general linear model (GLM) analysis. Moreover, competition performances were reported by presenting the players’ individual competition score curve during the two-and-a-half-year follow-up.

Then firstly, logistic regression analyses including the raw scores of the perceptuo-motor skills assessment items were used to examine if their outcomes predicted whether or not young players participated in the table tennis competition within the measurement period of this study. Secondly, a generalized estimating equations (GEE) analysis was conducted to explore the predictive value of the perceptuo-motor skills assessment items for the longitudinal competition outcomes of five competition periods in univariable and multivariable models (backward procedure). Test age (years), sex, and training experience (months) were included as covariates in both the logistic and GEE analyses. Alpha was set at 0.05 for significance for all analyses.

## Results

**Fig 1:**
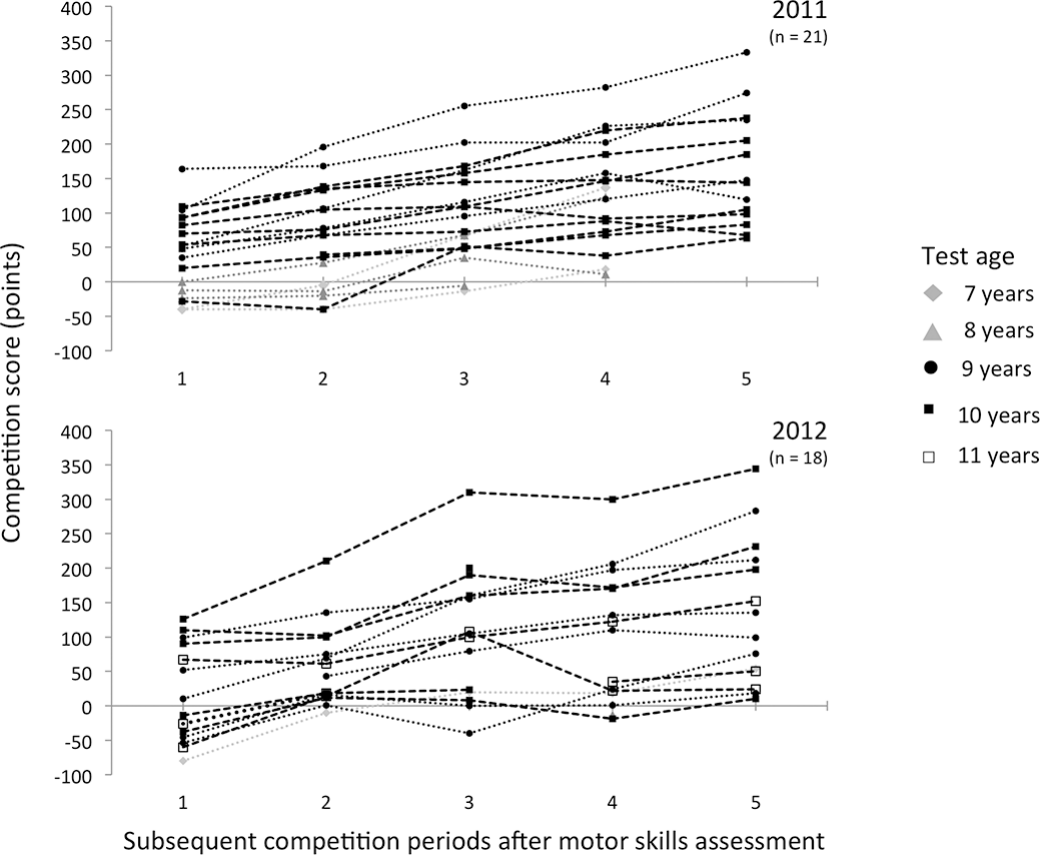
Competition score curve per player over five competition periods (= 2.5 years). Lines represent the individual players.

A total of 48 young table tennis players (7–11 years) participated in this study; 25 from the regional talent day in 2011 and 23 from the regional talent day in 2012. This number amounts to approximately 20–25% of the players available in this age category in the eastern department per year. The sample characteristics and descriptive results of the perceptuo-motor skills assessment and covariates are presented in Tables 1 and 2, respectively. All raw scores and the total score of the motor skills assessment for the total group were evaluated as normally distributed; means and medians were similar and the range around the mean followed a normal distribution. There were no missing data. The univariable GLM analysis for the total score demonstrated significant main effects of sex (F = 5.479, *p* = 0.024) and test age (F = 3.954, *p* = 0.008). Boys tended to perform better than girls on the test items, and older players had better scores than younger players. As such, sex and test age were depicted as covariates. No interaction effects were found. Fig 1 presents the competition score curve per player during the five consecutive competition periods after the perceptuo-motor skills assessment (n = 39). Nine players did not enter the competition during the two-and-a-half-year follow-up or before this study was conducted. Most competition players participated in all five consecutive competitions after the regional talent day (n = 25). The other competition players did not participate in all the competition periods (four periods n = 6, three periods n = 2, two periods n = 2, one period n = 4). The competition curves show that, in general, players tend to improve during the competition periods. Additionally, it shows that younger players (test age 7–8 years) have lower scores than their older counterparts (test age 9–11 years). All data obtained are available (S2 File).

**Table 1:** Characteristics of participants.

	Total group	Non competition	Competition
Total of participants (n)	48	9	39
Participants 2011 (n)	25	4	21
Participants 2012 (n)	23	5	18
Girls (n)	24	6	18
Boys (n)	24	3	21
Test age 7 years (n)	3	0	3
Test age 8 years (n)	6	2	4
Test age 9 years (n)	20	5	12
Test age 10 years (n)	17	1	16
Test age 11 years (n)	7	1	4
	M (range)	M (range)	M (range)
Test age (y)	9.3 (7–11)	9.1 (8–11)	9.4 (7–11)
Height (cm)	143 (126–161)	142 (129–156)	143 (126–143)
Weight (kg)	35 (22–57)	35 (26–57)	35 (22–56)
Training experience (months)	13 (1–48)	4 (1–6)	13 (2–36)
Current training (hours / week)	2.7 (1–7)	1.9 (1–3)	2.5 (1–7)

n = number, M = mean.

**Table 2:** Descriptive statistics perceptuo-motor skills assessment and logistic regression competition participation.

Variables	Total (n = 48)			Non competition (n = 9)			Competition (n = 39)			Logistic regression		
	M	SD	Range	M	SD	Range	M	SD	Range	B (SE)	OR (95% CI)	*p*
Sprint (s)	35	3.4	29–44	35	2.4	31–37	35	3.6	29–44	0.021 (0.112)	1.021 (0.820–1.271)	0.852
Agility (s)	25	4.5	18–45	27	3.9	21–35	25	4.6	18–45	-0.100 (0.076)	0.905 (0.780–1.051)	0.191
Vertical jump (cm)	31	5.6	17–44	29	4.5	19–35	31	5.9	17–44	0.059 (0.068)	1.060 (0.927–1.213)	0.391
Speed while dribbling (s)	24	6.3	15–43	25	5.5	20–37	24	6.6	15–43	-0.026 (0.056)	0.975 (0.874–1.088)	0.648
Aiming at target (points)	22	9.8	4–42	19	6.7	8–26	23	10.3	4–42	0.048 (0.040)	1.049 (0.969–1.135)	0.237
Ball skills (points)	19	6.4	5–31	17	8.2	5–29	19	6.0	5–31	0.064 (0.059)	1.066 (0.949–1.197)	0.280
Throwing a ball (m)	9	1.4	7–12	9	1.2	7–11	9	1.5	7–12	0.263 (0.254)	1.301 (0.776–2.182)	0.318
Eye-hand coordination (points)	13	7.0	0–26	11	4.5	2–18	13	7.5	0–26	0.053 (0.053)	1.055 (0.950–1.171)	0.317
Total score (percentiles)	466	143	160–720	398	102	190–520	481	147	160–720	-	-	-
Age at test moment (y)	9.3	1.0	7–11	9.1	0.9	8–11	9.4	1.1	7–11	0.231 (0.355)	1.260 (0.628–2.529)	0.515
Sex	-	-	-	-	-	-	-	-	-	-0.847 (0.777)	0.429 (0.094–1.964)	0.275
Training experience (months)	11	9.0	1–36	4	1.9	1–6	13	9.1	2–36	0.421 (0.167)	1.523 (1.097–2.115)	0.012^*^

n = number, M = mean, SD = standard deviation, B = regression coefficient, SE = standard error, OR = odds ratio, CI = confidence interval

*p <0.05.

Univariable logistic regression analyses to show whether the assessment’s results predicted competition participation within the measurement period of this study demonstrated no significant predictors; all regression coefficients show *p* >0.05 with the odds ratio 95% confidence intervals including 0 (n = 48; Table 2). Moreover, the differences between the means of the non-competition (n = 9) and competition players (n = 39) at the individual test items (Table 2) were generally smaller than the standard error of the measurements of the items found in the previous studies focusing on reproducibility [35,36]. Only the covariate training experience presented to be a significant predictor for future competition participation (*p* = 0.012). This implies that players with more training experience at the moment of testing are more likely to join competition in the future. Since no other univariable models were found, multivariable models are not proposed.

The data of 39 young competition players could be included in the GEE analyses (Table 3). All perceptuo-motor skills assessment’s test items except one (agility), test age, and training experience predicted significantly the longitudinal competition outcome in univariable models (*p* <0.001). The best fitting multivariable model using a backward procedure (R^2^ = 51%) included the test items: aiming at target, throwing a ball, and eye-hand coordination. Although test age did not have a significant contribution to this best fitting model, inclusion was proposed as this factor showed to be a possible confounder in this study and to have influenced the item scores in previous studies [27,35,36]. Consequently, another multivariable model was suggested for improved estimations of the test items’ regression coefficients. This model explained 53% of the variance of the longitudinal competition results, including 5 competition periods in 2.5 years.

**Table 3:** Predictive validity results for competition performance (univariable and multivariable generalized estimated equations analyses, n = 39).

Variables	B (SE)		
	Univariable	Best fitting	Multivariable
(Intercept)	-	-175.597** (46,50)	-93.588 (66,29)
Sprint (s)	-10.109** (2.30)	-	-
Agility (s)	-6.055 (3.92)	-	-
Vertical jump (cm)	4.401** (1.08)	-	-
Speed while dribbling (s)	-6.854** (1.33)	-	-
Aiming at target (points)	4.642** (0.92)	2.169* (0.89)	2.010** (0.82)
Ball skills (points)	6.535** (1.22)	-	-
Throwing a ball (m)	35.255** (4.99)	14.554** (5.61)	19.562** (5.71)
Eye-hand coordination (points)	7.300** (0.86)	4.787** (1.04)	5.510** (1.08)
Age at test moment (y)	3.103** (0.59)	-	-1.123 (0.66)
Sex	38.357 (22.97)	-	-
Training experience (months)	2.584** (0.60)	-	-
R^2^		51%	53%

B = regression coefficient, SE = standard error

* *p* <0.05

** *p* <0.01

## Discussion

This study focused on the capacity of the perceptuo-motor skills assessment in young table tennis players (7–11 years) to predict future competition participation and competition performance over a period of two-and-a-half year. The results of this study demonstrated that the perceptuo-motor skills assessment outcomes did not predict competition participation within the measurement period of five competitions after the assessment. Competition participation might be better explained by other factors than perceptuo-motor skills, e.g. motivation, parental influences, training facilities, and the availability and level of team members, as proposed in the introduction [38]. Nevertheless, the results did indicate that the assessment of underlying perceptuo-motor skills may be an important predictor for future competition performance in young competition players (7–11 years) of the regional talent day over a period of 2.5 years. Based on our previous research, it even seems that future competition performance is better explained by the perceptuo-motor skills assessment than table tennis performance at the moment of testing [35]. The explained variance shown in the current study for the prediction of the longitudinal competition results (R^2^ = 51–53%) is higher than those presented in the previous study for the association between the perceptuo-motor skills assessment and national ranking at the moment of testing (boys R^2^ = 29%, girls R^2^ = 17%, 6–10 years) [35]. This supports the idea that the underlying perceptuo-motor skills reflect the potential of young table tennis players, but also that other factors are of influence [4]. Moreover, it needs to be taken into account that competition scores are used as performance outcomes in this study and ranking position was used in the previous study [35]. Nevertheless, based on the results of this study, the perceptuo-motor skills assessment seems a suitable instrument as a part of talent development programmes including selection procedures and the monitoring of young players.

To our knowledge, there are no recent prospective studies, which include perceptuo-motor skills to predict table tennis sport performance. In other racquet sports, only two studies were found on this topic. Elliott, Ackland, Blanksby and Bloomfield (1990) and Panjan, Sarabon and Filipčič (2010) used an observational design to predict tennis performance from anthropometric, physiological and perceptuo-motor indicators [43,44]. Comparable to our results, perceptuo-motor test items were considered the best predictors for performance. However, the predictive value in these studies was difficult to determine. Elliot, Ackland, Blanksby and Bloomfield (1990) included only group comparisons regarding playing level at different age groups (11, 13 and 15 years). No prediction models were presented for future performance. Moreover, the accuracies of Panjan, Sarabon and Filipčič their models (2010) for the competition performance at the age category of 12–16 and over the age of 16, based on both the assessment under the age of 12 and 12–16 years, respectively, were poor with high relative absolute errors (0.59–0.99) [43,44]. Consequently, this study is one of the first attempts at forecasting performance curves in table tennis or other racquet sports on the basis of perceptuo-motor skills showing significant predictors.

The results of the current study correspond with the findings of Vandorpe, Vandendriessche, Vaeyens, Pion, Lefevre, Philippaerts et al. (2013) [7]. Their study included young gymnasts (7–8 years), in which the potential to become an elite gymnast was also considered to depend on perceptuo-motor skills. Vandorpe, Vandendriessche, Vaeyens, Pion, Lefevre, Philippaerts et al. (2013) showed that non sport-specific perceptuo-motor tests were significant predictors for performance results in young elite gymnasts, explaining more than 40% of the competition outcome two years later. In this study, the ‘Körperkoordinations Test für Kinder’ was used to measure general perceptuo-motor skills in children. The test items of this test battery appeal to a great extent on a child’s ability to maintain balance in dynamic situations, which is in line with the specific demands of gymnastics. In a parallel line, the test items aiming at target, throwing a ball, and eye-hand coordination used in the current study appeal to the players’ ability to control a ball [35], which is a specific requirement for table tennis. Moreover, the study of Pion, Fransen, Deprez, Segers, Vaeyens, Philippaerts et al. (2015) revealed that tests assessing perceptuo-motor skills, which were non-specific to volleyball, discriminated between elite and sub elite adolescent volleyball players (p <0.036). The authors conclude that volleyball as a skill-based sport requires a well-developed level of perceptuo-motor skills [45]. Consequently, it is suggested that perceptuo-motor skills indeed play an important role in the development of young athletes’ performance level in sports consisting of complex motor tasks. Yet, specific tests to assess the underlying skills inherent to a particular sport seem appropriate.

Although the relation between perceptuo-motor skills and future performance is highlighted by the current study and earlier results [7,43,44], the multidimensionality of talent development must be taken into account [1]. The perceptuo-motor skills assessment only measures potential in the perceptuo-motor domain. The variance of the competition outcomes that could not be explained is probably due to other factors. Mental aspects, like motivation, self-efficacy, volition, and self-esteem, and contextual factors, such as training facilities and parental support, are hypothesized to have a substantial influence on performance outcome [46,47]. This probably also accounts for the prediction of future competition participation. As hypothesized, personal or contextual factors other than perceptuo-motor skills seem more important to young players and their parents to decide to participate in competitions. Therefore, the perceptuo-motor skills assessment as a part of a talent development programme, should be accompanied by an evaluation and monitoring of other critical factors.

Some limitations of this study need to be acknowledged. First of all, this study included a small sample recruited during the regional talent day and already selected by club trainers. Although the perceptuo-motor skills assessment is used during regional talent days, the generalisation of the findings to the players on the national talent day, including players from other regions, is worth discussing. Since no ceiling effect seems to exist with regard to the test item scores, the total score, and the competition outcomes in this study and sufficient variety in the scores is expected between players participating in the national talent day, it is hypothesized that generalization is possible. The predictive validity should be studied in a larger sample, including players from the national talent day to verify this hypothesis. The assessment might lose its strength in a small homogenous sample regarding perceptuo-motor skills, e.g. the national top 10 players. Evaluating other factors such as motivation, trainability, and mental aspects might be more sensible in such context. Secondly, only a relatively short follow-up of 2.5 years was realised in this study. This implies that the perceptuo-motor skills assessment’s results are used to predict performance results still at a relative young age. Although, significant predictors were found, these results do not guarantee the predictive value of the perceptuo-motor skills assessment over a longer time period or in other age-ranges. A longer follow-up period is crucial for evaluating the solidness of the predictors for adult success. The inclusion of repeated measurements to monitor the players’ development during the follow-up might improve prediction accuracy, as a longer period of time increases the interference of other factors. Thirdly, to optimize the power of this study, analyses were conducted using the total sample. Results from boys and girls within the test age spanning from 7 to 11 years were analysed all together. Consequently, the analyses included different developmental stages in which the associations were calculated, especially in considering influence of differences in growth and maturation [48]. Although sex and test age were included as covariates, it is recommended for future research to split the analyses for boys and girls as differences in predictors might exist and reduce the age span for prediction models.

In conclusion, we found that the predictive validity of the perceptuo-motor skills assessment has promising prospects. An instrument such as the perceptuo-motor skills assessment can objectify a young player’s potential and, for that reason, support selection decisions by coaches. It has to be emphasized, however, that talent development is a multidimensional process [1], and the perceptuo-motor skills assessment is suitable for being a part of a talent development programme. Moreover, to interpret individual player’s test scores, it is important that the player’s sex, test age, training experience, growth and maturity level are taken into account [48,49]. Furthermore, talent development programmes do not intend to limit the freedom of choice in children to practice a particular sport. Trainers and coaches should also be aware of the potential risks of early specialization and selection (e.g. injuries, mental exhaustion and drop-outs) [50,51]. The perceptuo-motor skills assessment is only intended to identify those children excelling in the perceptuo-motor skills essential for table tennis. Finally, additional studies are needed to evaluate the predictive value in a larger sample over a longer period of time.

## Supporting Information

S1 File Test protocol perceptuo-motor skills assessment. (PDF)

S2 File Full dataset. (PDF)
