# Supplementary material for: Can Perceptuo-Motor Skills Assessment Outcomes in Young Table Tennis Players (7–11 years) Predict Future Competition Participation and Performance? An Observational Prospective Study
Source: PLoS One. 2016 Feb 10;11(2):e0149037. doi: 10.1371/journal.pone.0149037 (PMC4749309; doi:10.1371/journal.pone.0149037)
Supplement: S1 File — (PDF) [file pone.0149037.s001.pdf]

Full protocol perceptuo-motor skills assessment

Supplementary File

Test protocol 'Sprint'

Description
Test duration: 2-5 minutes per player
Assessor: 1, the assessor should be familiarized with the protocol and instructed by an expert assessor.

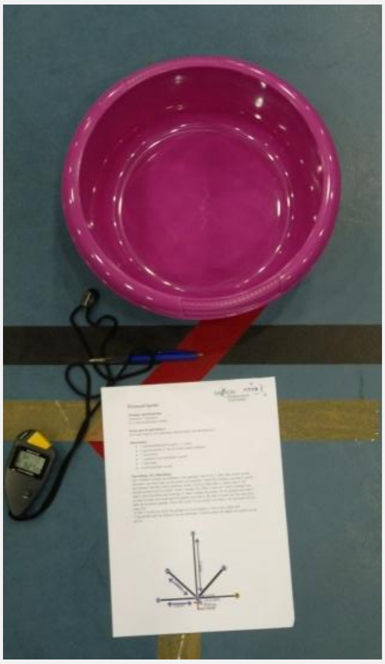

Materials:

- 5 table tennis balls (4 white balls and 1 yellow/orange ball)
- 6 round trays with numbers (Ø 28 cm)
- Stopwatch
- Measuring tape (5 m)
- Tape to mark distances
- Score form
- Pencil

Set-up:
The player must be tested in an open area. On the ground, the locations of the trays 6 are marked with tape (see Figure 1), and the trays are placed in a triangle. Optionally, the trays can be taped to the floor. The trays are numbered from 0 to 5.
At tray 0 a start / finish line is taped; this line is placed 10 cm before tray 0 and is 40 cm long. In each tray 1 to 4 one white ball is placed, yellow / orange ball is placed in the tray 5.

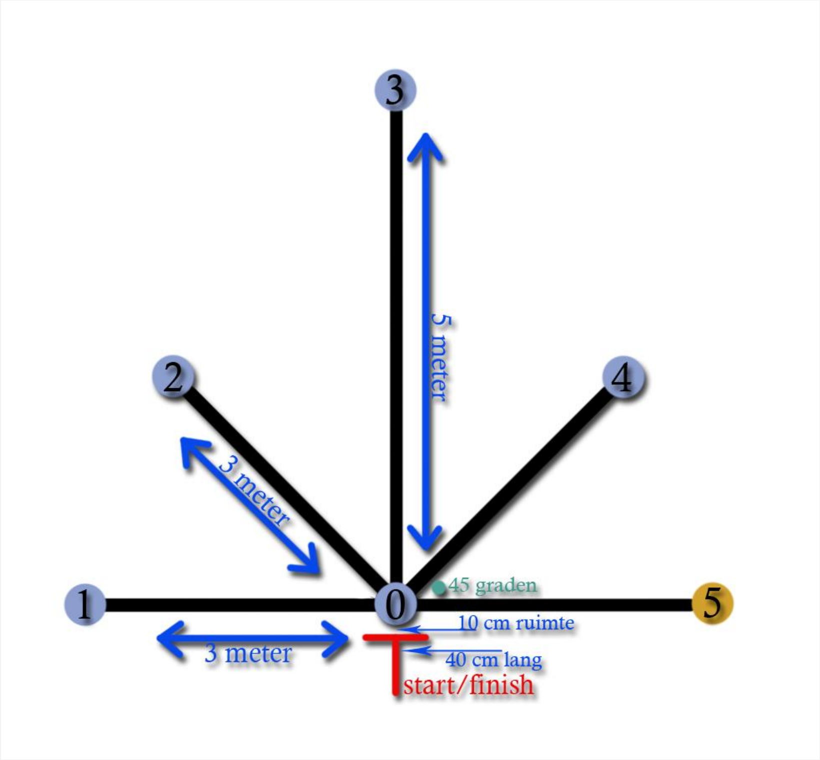

Figure 1: Test set-up

Assignment:
The player tries to sprint through the course as quickly as possible. He / she starts at the start / finish line right in front of tray 0. After the agreed starting signal, the player gets the first ball from tray 1 and brings it to 0. Then he / she continues by doing the same for the balls in tray 2 to 5. Once the yellow / orange ball from tray 5 has been put in tray 0, the player picks up a white ball and brings it back to tray 1. Then the same for the other balls; the player returns them to the trays one by one. The time stops when all balls are put back into the trays and the player crosses the start / finish line. Each player gets two attempts. If there are more players, make sure all players finish their first attempt after that everyone goes for the second attempt. If there is only one player, make sure he / she has some period (minimal 3 minutes) to recover.

Demonstration:
Emphasize during the instruction / demonstration of the assignment the following:

- The test attempt only starts after the test taker gave the start signal.
- Sprint as fast as possible.
- Do not sprint around the trays.
- Place the balls in the trays; you are not allowed to throw.
- Count in your head so you will not forget at which tray you should go.
- When balls are lost, the player needs to retrieve the ball this as soon as possible by him-/herself. Try to prevent this as it takes time.
- Encouragements and corrections are only allowed during the practice phase, NOT during the test attempt.

Practice phase:
There is no practice phase. However, ensure the players did a warming-up before the testing starts.

Procedural errors:
When the player or the assessor make a mistake during the execution of the test or if there is a disturbing factor in the environment, we speak of a procedural error. When a procedural error happens the attempt must be terminated immediately and will not count in the score. The player may perform the test attempt again after a reasonable period for recovery. However, when the procedural error is made because of 'incapability', the attempt is considered normal completed by the player.

Score:
The time of two attempts is recorded with the accuracy of one hundredth of a second (e.g. 23.58 sec.).

Registration:
The times of two attempts are recorded on the score sheet. The best attempt is used in further analysis.

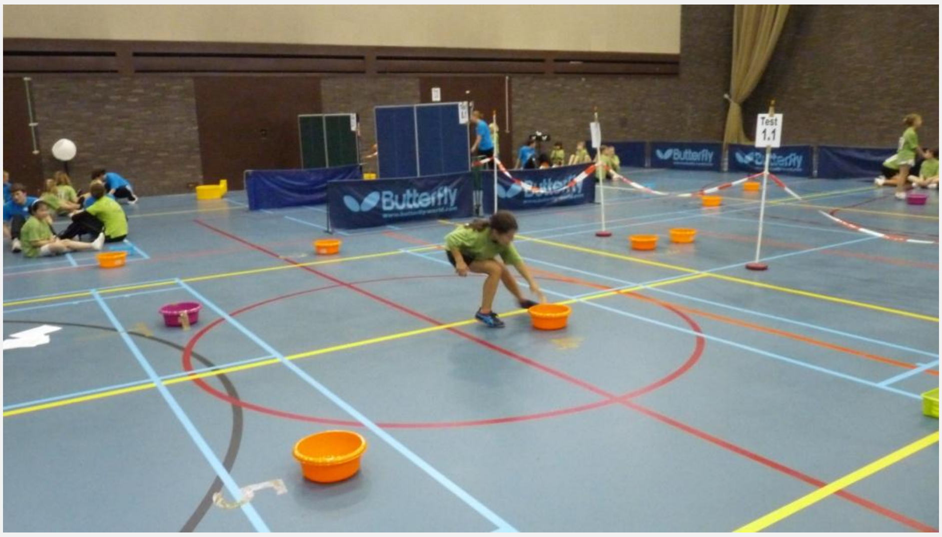

Test protocol 'Agility'

Description
Test duration: 2-3 minutes per player
Assessor: 1, the assessor should be familiarized with the protocol and instructed by an expert assessor.

Materials:
- 1 gymnastic cabinet (height 110 cm)
- 2 setups to crawl underneath and stepping over them (height 51 cm)
- 4 gymnastic floor mats (100 x 150 x 7 cm)
- Stopwatch
- Measuring tape (5 m)
- Tape to mark distances
- Score form
- Pencil

Set-up:
The player must be tested in an open area. Place the gymnastic cabinet and gymnastic floor mats according to photo 1. Put the set-ups at 2 meter distance of the middle of the gymnastic cabinet. The height of the set-ups must be 51 cm from the ground while excluding the thickness of the gymnastic floor mat. Mark the start line at 1 meter distance of the gymnastic cabinet.

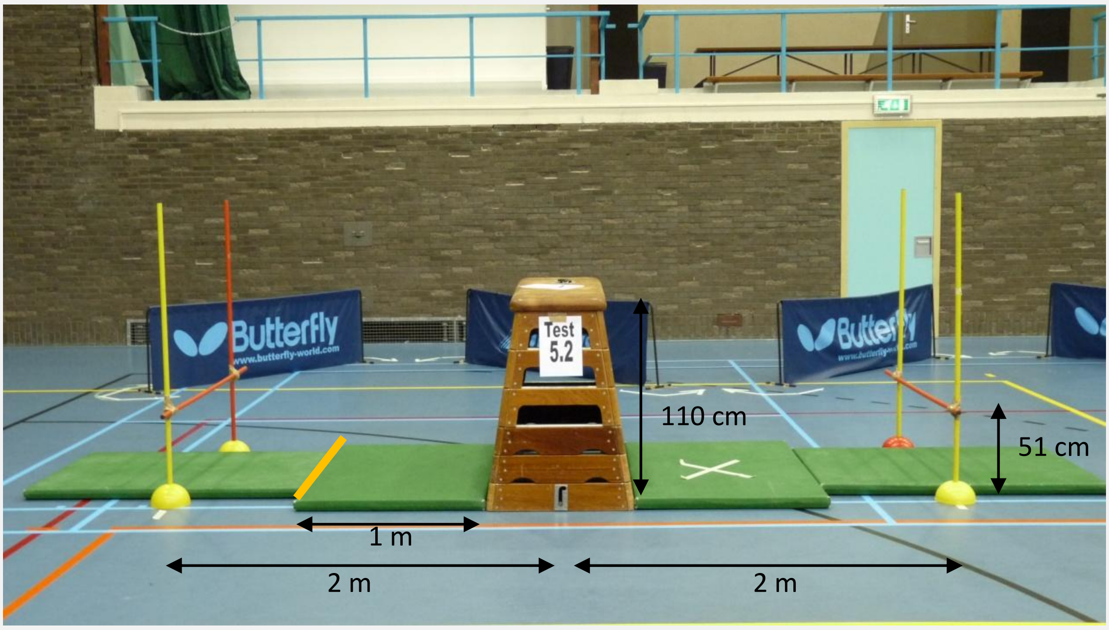

Photo 1: Test set-up

Assignment:
The player tries to sprint through the course as quickly as possible. He / she starts before the start line standing straight up and facing the gymnastic cabinet. After the agreed starting signal, the player climbs over the cabinet, crawls under the set-up and directly steps over the set-up again in the direction of the cabinet. The player continues to do so till he / she had climbed over the gymnastic cabinet for the 5th time. The time stops when the player hits the floor mat with both feet. The assessor counts out loud the number of climbs over the gymnastic cabinet. Each player has 1 attempt.

Demonstration:
Emphasize during the instruction / demonstration of the assignment the following:
- The test only starts after the assessor gave the start signal.
- Climb and crawl as fast as possible.
- Listen carefully to the assessor how many time you climbed over the cabinet.
- Encouragements and corrections are only allowed during the practice phase, NOT during the test attempt.

Practice phase:
The players may practice the course one time, which includes two climbs over the gymnastic cabinet. There is no need to rush during practice. Ensure the players did a warming-up before the test starts.

Procedural errors:
When the player or the assessor make a mistake during the execution of the test or if there is a disturbing factor in the environment, we speak of a procedural error. Examples of procedural errors are taking a wrong route through the course or falling. When a procedural error happens the attempt must be terminated immediately and will not count in the score. The player may perform the test attempt again after a reasonable period for recovery. However, when the procedural error is made because of 'incapability', the attempt is considered normal completed by the player.

Score:
The time of two attempts is recorded with the accuracy of one hundredth of a second (e.g. 23.58 sec.).

Registration:
The time of the one attempt is recorded on the score form.

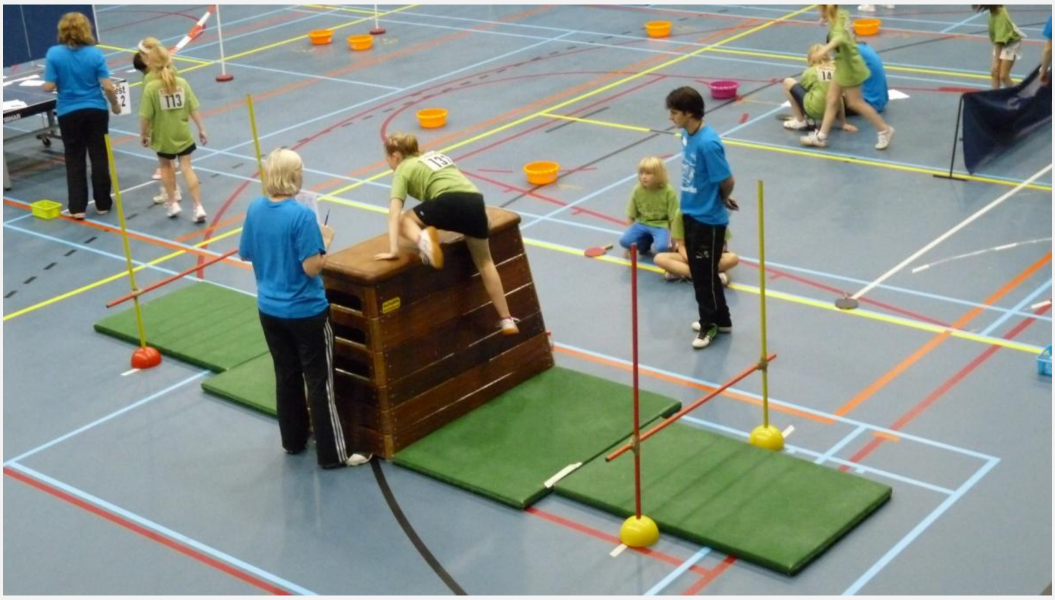

Test protocol 'Vertical jump'

Description
Test duration: 2-3 minutes per player
Assessor: 1, the assessor should be familiarized with the protocol and instructed by an expert assessor.

Materials:

- Wall
- Magnesium powder
- Measuring tape (2 m)
- Tape to fix the tape to the wall
- Score form
- Pencil

Set-up:
The player must be tested in an open area. Mark a distance of 10 cm to the wall on the floor (Photo 1).

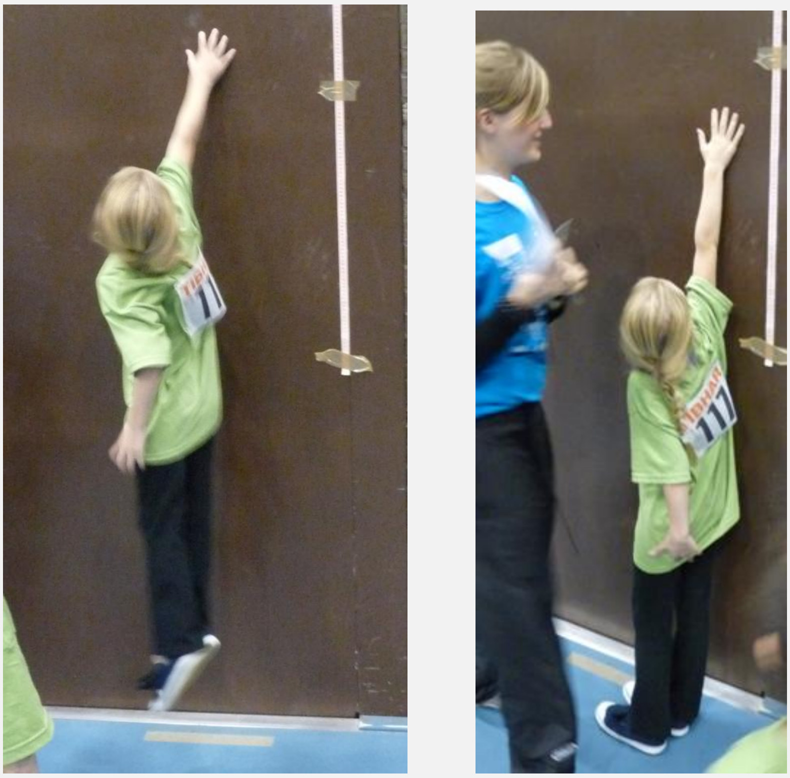

Photo 1: Test set-up

Assignment:
The player tries to jump and tick the wall with one hand as high possible. Each player has 3 attempts. Before jumping the player need to put some magnesium powder on his / her fingers. At starting position the players stands next to the wall while facing parallel to the wall. The three attempts are conducted directly after each other. After jumping the players needs to stand similar to the starting position with the arm that is closest to the wall held straight up touching the wall (Photo 1, right).

Demonstration:
Emphasize during the instruction / demonstration of the assignment the following:

- Jump as high as possible.
- Start with both feet flat on the ground.
- Encouragements and corrections are only allowed during the practice phase, NOT during the test attempt.

Practice phase:
There is no practice phase.

Procedural errors:
When the player or the assessor make a mistake during the execution of the test or if there is a disturbing factor in the environment, we speak of a procedural error. Examples of procedural errors are taking a wrong route through the course or falling. When a procedural error happens the attempt must be terminated immediately and will not count in the score. The player may perform the test attempt again after a reasonable period for recovery. However, when the procedural error is made because of 'incapability', the attempt is considered normal completed by the player.

Score:
For all three attempts the differences between the jump height and starting position with the arm help straight up are calculated in cm.

Registration:
The distances of the three attempts are recorded on the score sheet. The best attempt counts for further analysis.

Made by the Netherlands Table Tennis Association – Irene Faber The Netherlands, November 2012

Test protocol ‘Speed while dribbling’

Description
Test duration: 2-3 minutes per player
Assessor: 1, the assessor should be familiarized with the protocol and instructed by an expert assessor.

Materials:

- Tape to mark start/finish and route
- 4 table tennis fences (2.30 m)
- 1 basketball (size 5)
- Stopwatch
- Measuring tape (15 m)
- Goniometer
- Score form
- Pencil

Set-up:
The player must be tested in an open area. Place the table tennis fences in an angle of 90 degrees relative to each other. In total, the total distance of the course should be 11 meters. Between two fences must be a distance of one meter. At 1 meter from the first partition is set a start and finish line. Mark the route with tape (or chalk).

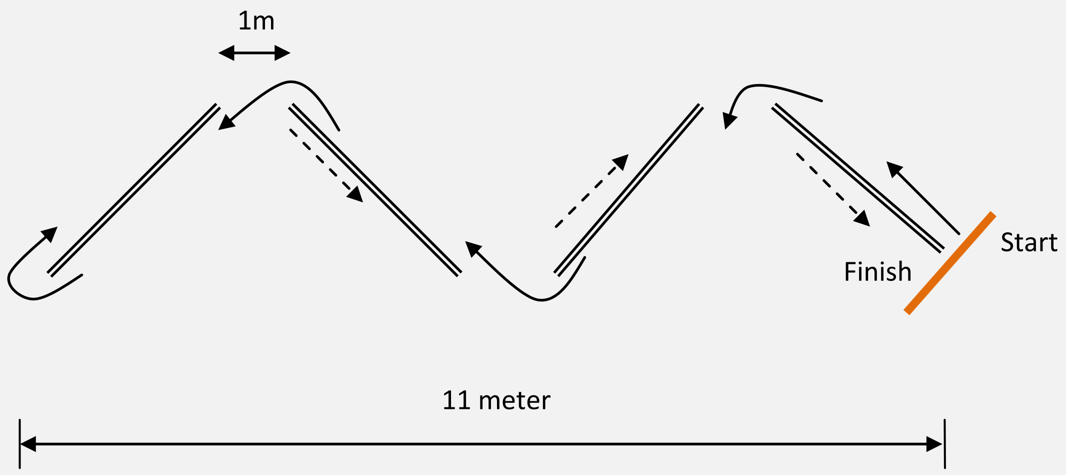

Figure 1: Test set-up

Assignment:
The player tries to dribble through the course as quickly as possible. He / she starts at the start line holding the ball in both hands. After the agreed starting signal, the player zigzags side-wards through the course facing to the table tennis fences at all times. Only dribbling with one hand is allowed, although switching hands is permitted. The time stops when the player crosses the finish line. Each player has one attempt.

Demonstration:
Emphasize during the instruction / demonstration of the assignment the following:

- The test only starts after the assessor gave the start signal.
- Zigzag as fast as possible.
- Always move side-wards facing the table tennis fences.
- Dribble with one hand at the time.
- When you lose the ball, get it back as quick as possible and continue where you dropped off.
- Encouragements and corrections are only allowed during the practice phase, NOT during the test attempt.

Practice phase:
During the practice phase the course is practiced without ball to familiarize the players with the route. Players are instructed to move side-wards while facing the table tennis fences.

Procedural errors:
When the player or the assessor make a mistake during the execution of the test or if there is a disturbing factor in the environment, we speak of a procedural error. When a procedural error happens the attempt must be terminated immediately and will not count in the score. The player may perform the test attempt again after a reasonable period for recovery. Such an extra attempt is only given once. Examples of procedural errors are:

- If a player losses the ball often (>2 times) or needed to get the ball from a long distance.
- If a player takes a wrong route through the course.
- If the players dribbles with both hands.

However, when the procedural error is made because of 'incapability', the attempt is considered normal completed by the player.

Score:
The time of the attempt is recorded with the accuracy of one hundredth of a second (e.g. 23.58 sec.).

Registration:
The time of the attempt is recorded on the score sheet. In case of two attempts, the best time counts.

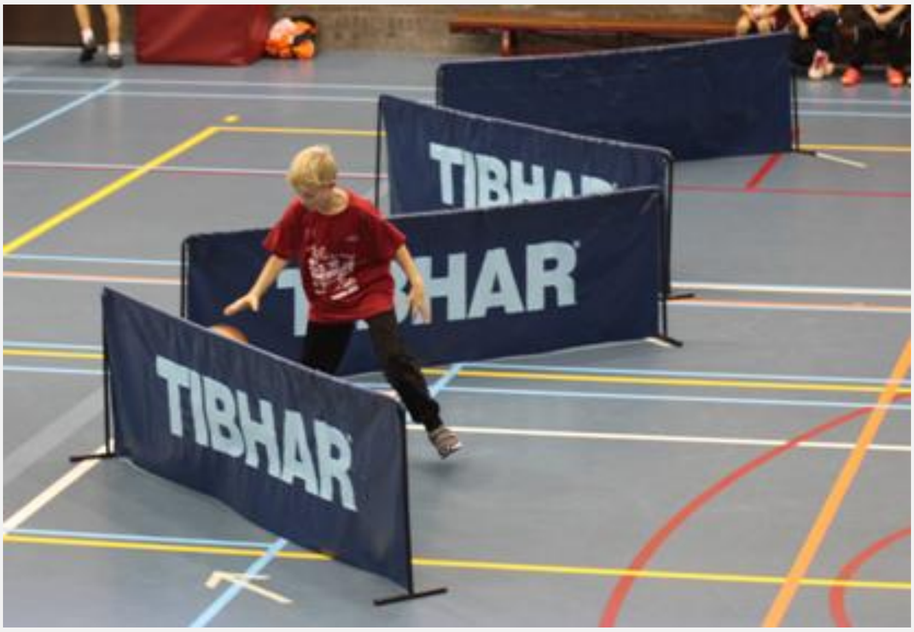

Made by the Netherlands Table Tennis Association – Irene Faber The Netherlands, November 2012

Test protocol 'Aiming at target'

Description
Test duration: 3-4 minutes per player
Assessor: 1, the assessor should be familiarized with the protocol and instructed by an expert assessor.

Materials:
- 14 table tennis balls
- Tray for the table tennis balls
- 1 standard table tennis racquet (for novice players)
- Target (outer circle Ø 60 cm, inner circle Ø 20 cm)
- Tape measure
- Tape to mark 2,5 meter distance
- Score form
- Pencil

Set-up:
The player must be tested in an open area. The space needed is approximately 2 x 3 meters, which should be surrounded by table tennis fences. Measure a distance of 2.5 meter to the central point in the target and mark this with a piece of tape (Picture 1). Next to the starting point (X) the tray with table tennis balls must be placed. Ensure that the player has to step aside from the starting point to grasp a new ball for each attempt; the player has to orientate each time to the target.

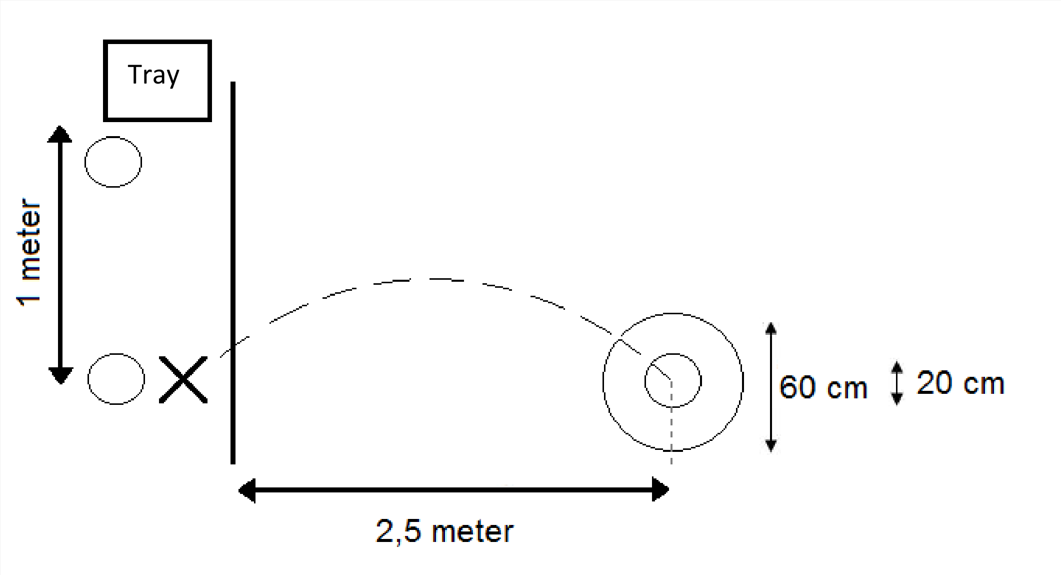

Picture 1: Top view of test set-up

Assignment:
The player must aim for the inner circle of the target using the standard racquet while standing at the starting point at 2.5 meter distance. Hitting the inner circle is awarded with 6 points, hitting the outer circle with 4 points. Each player has 10 attempts during which forehand and backhand strokes must be alternated. The assessment always starts with the forehand. After each attempt, the players grasps a new ball out of the tray and takes his / her starting position straight in front of the target. The player may choose his / her preferred hand for this assessment.

Demonstration:
The assessor demonstrates the task during the instruction. Emphasize during the instruction / demonstration of the assignment the following:
- Aim for the inner circle of the target.
- Stand upright behind the mark of 2.5 meter distance. The shoulders must also be behind the marking line.
- Pay attention on the alternation of forehand and backhand.
- Encouragements and corrections are only allowed during the practice phase, NOT during the test attempt.

Practice phase:
Each player has 4 attempts to practice; 2 forehand and 2 backhand. Directly after these attempts the assessment starts.

Procedural errors:
When the player or the assessor make a mistake during the execution of the test or if there is a disturbing factor in the environment, we speak of a procedural error. When a procedural error happens the attempt must be terminated immediately and will not count in the score. The player may perform the test attempt again. However, when the process error is made because of 'incapability', the attempt is considered normal completed by the player. Examples of this are:

- The players wasn't forced to orientate himself / herself again to the target.
- The player does not stand behind the marking line of 2.5 meter.
- The player played with his forehand when backhand should be used or vice versa.

As an assessor, always be alert to possible misunderstanding of the player. Be aware that procedural errors are not used as extra training.

Score:
The points of every attempt is noted (inner circle = 6, outer circle = 4, no hit = 0).

Registration:
The sum of the 10 attempts is calculated as final score.

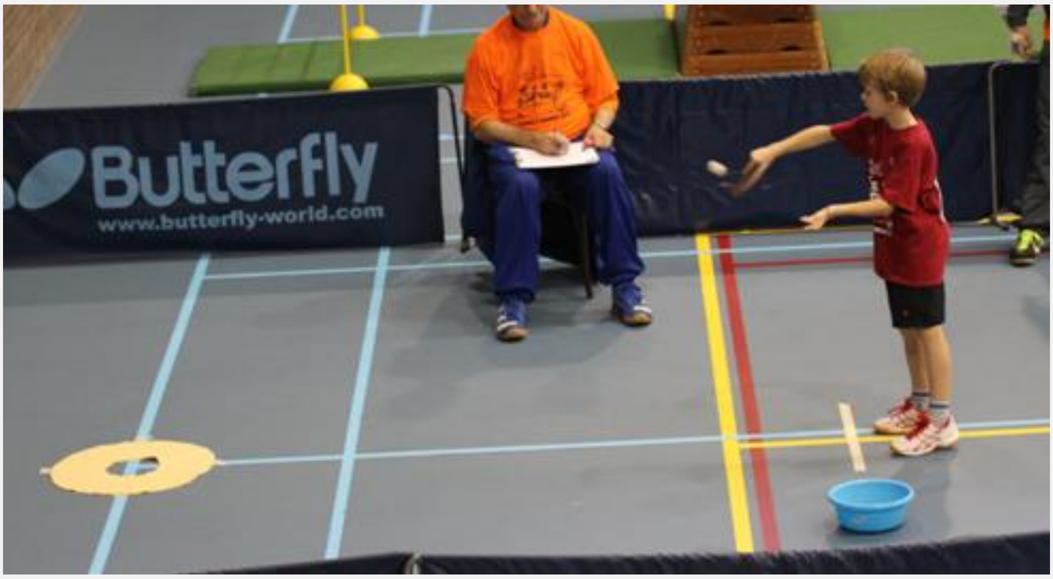

Made by the Netherlands Table Tennis Association – Irene Faber The Netherlands, November 2012

Test protocol 'Ball skills'

Description
Test duration: 3-4 minutes per player
Assessor: 1, the assessor should be familiarized with the protocol and instructed by an expert assessor.

Materials:

- 26 table tennis balls
- 1 table tennis table halve which can be placed vertically
- Tray for the table tennis balls
- Target (outer circle Ø 75 cm, inner circle Ø 33.5 cm)
- Tape measure
- Tape to mark distances
- Score form
- Pencil

Set-up:
The player must be tested in an open area. The space needed is approximately 3 x 4 meters. A ground map of the test set up is shown in Picture 1. This ground map is for right handed players. For left handed players the set-up must be mirrored.

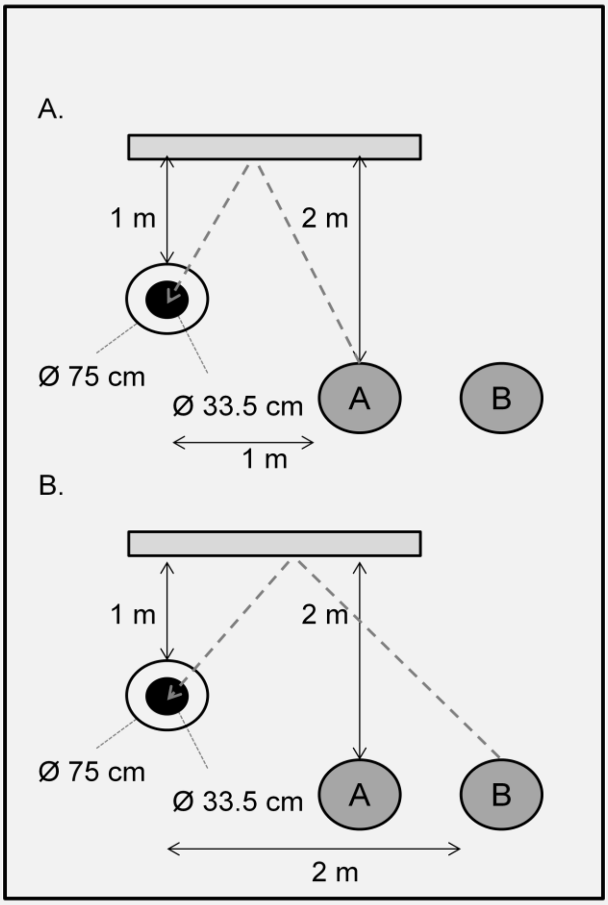

Picture 1: Top view of test set-up (right-handed players)

Assignment:
The player must aim for the inner circle of the target by throwing the table tennis ball via the vertical positioned table tennis table. The players start the first attempt at position A (picture 1A) and for the second attempt the player throws from position B (picture 1B) and so on. Hitting the inner circle is awarded with 2 points, hitting the outer circle with 1 points. Each player has 20 attempts at which position A and B must be alternated. The assessment always starts at A. The player may choose his / her preferred hand for this assessment.

Demonstration:
The assessor demonstrates the task during the instruction. Emphasize during the instruction / demonstration of the assignment the following:

- Aim for the inner circle of the target via the vertical table tennis table.
- Stand upright behind at the right position.
- Pay attention on the alternation of position A and B.
- The ball may not bounce on the floor before hitting the target. If it does bounce on the floor, that attempt does not yield points.
- Encouragements and corrections are only allowed during the practice phase, NOT during the test attempt.

Practice phase:
Each player has 6 attempts to practice; 3 at position A and 3 at position B. Directly after these attempts the assessment starts.

Procedural errors:
When the player or the assessor make a mistake during the execution of the test or if there is a disturbing factor in the environment, we speak of a procedural error. When a procedural error happens the attempt must be terminated immediately and will not count in the score. The player may perform the test attempt again. However, when the process error is made because of 'incapability', the attempt is considered normal completed by the player. Examples of this are:

- The player does not stand behind the marking line at the starting position.
- The player threw alongside, over or under the table tennis table.

As an assessor, always be alert for possible misunderstanding of the player. Be aware that procedural errors are not used as extra training.

Score:
The points of every attempt is noted (inner circle = 2, outer circle = 1, no hit = 0).

Registration:
The sum of the 20 attempts is calculated as final score.

Made by the Netherlands Table Tennis Association – Irene Faber The Netherlands, November 2012

Test protocol ‘Throwing a ball’

Description
Test duration: 2-3 minutes per player
Assessor: 1, the assessor should be familiarized with the protocol and instructed by an expert assessor.

Materials:
- 5 table tennis balls
- Tray for the table tennis balls
- Tape measure (25 meter)
- Tape to mark starting position
- Score form
- Pencil

Set-up:
The player must be tested in an open area. The space needed is 6 x 15-20 meters, which should be surrounded by table tennis fences. A ground map of the test set up is shown in Picture 1.

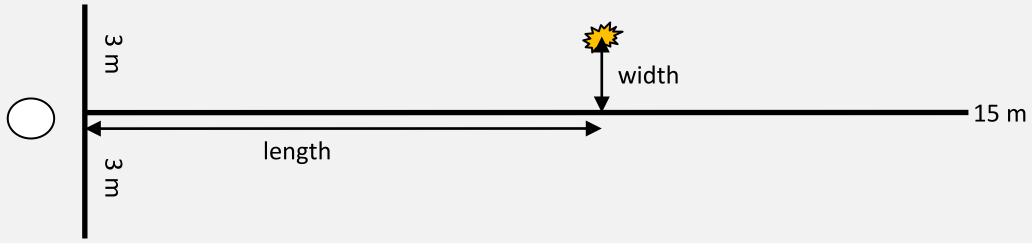

Picture 1: Top view of test set-up

Assignment:
The player takes place behind the marked line at the white dot (Picture 1). The player is instructed to throw a table tennis ball straight ahead and as far as possible. Each player has 3 attempts. The player may choose his / her preferred hand for this assessment.

Demonstration:
The assessor demonstrates the task during the instruction. Emphasize during the instruction / demonstration of the assignment the following:
- Throw as far as possible.
- Stand behind the mark while throwing.
- Don’t throw too high, to ensure optimal distance.
- Throw straight ahead.
- Encouragements and corrections are only allowed during the practice phase, NOT during the test attempt.

Practice phase:
There is no practice phase.

Procedural errors:
When the player or the assessor make a mistake during the execution of the test or if there is a disturbing factor in the environment, we speak of a procedural error. When a procedural error happens the attempt must be terminated immediately and will not count in the score. The player may perform the test attempt again. However, when the process error is made because of 'incapability', the attempt is considered normal completed by the player. Examples of this are:
• The player does not stand behind the marking line while throwing.
• The player throws the ball beyond the borders or at the ceiling.
As an assessor, always be alert to possible misunderstanding of the player. Be aware that procedural errors are not used as training.

Score:
The length and width (Picture 1) of all three attempts are noted in m (e.g. 7.4 m).

Registration:
The diagonal is calculated after the assessment. The best diagonal distance of three attempts is used as final score.

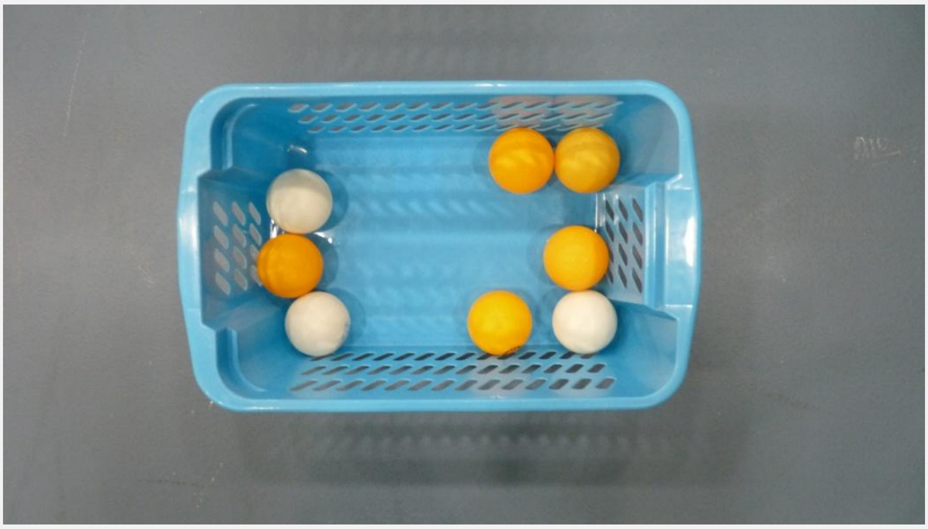

Made by the Netherlands Table Tennis Association – Irene Faber The Netherlands, November 2012

Test protocol ‘Eye hand coordination’

Description
Test duration: 2-5 minutes per player
Assessor: 1, the assessor should be familiarized with the protocol and instructed by an expert assessor.

Materials:
- 2 table tennis tables halves including 1 that can be positioned vertically
- 6 table tennis balls
- Stopwatch (timer counting backwards from 30 to 0 seconds)
- Measuring tape
- Tape to mark 1 meter distance
- Score form
- Pencil

Set-up:
The player must be tested in an open area. Make a rectangle field of 4 by 3 meters using a vertical positioned table tennis table placed a normal position table half and table tennis fences (see Photo 1). Measure a distance of 1 meter to the vertical table and mark this with a piece of tape (photo 2).

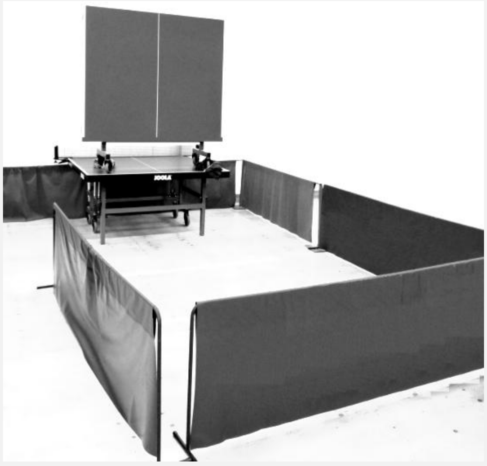

Photo 1: Test set-up

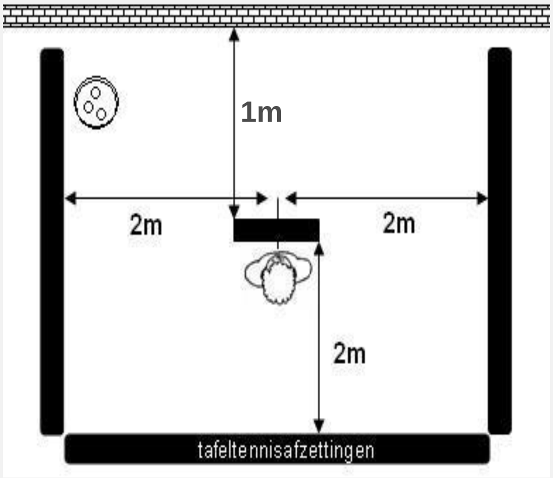

Photo 2: Top view of test set-up

Assignment:
The player takes place behind the 1-meter line tape. The player throws the ball to his / her own preference underhand or overhand with the right hand against the vertical table and catches it with the left hand without the ball touching the floor. Then the same using the left hand to throw and the right hand for catching. This is repeated for 30 seconds. The number of correctly caught balls is counted. The player has two test attempts. Scores of both attempts are noted, however, only the best attempt counts as final outcome.

Demonstration:
Emphasize during the instruction / demonstration of the assignment the following:
- The test only starts after the assessor gave the start signal.
- Stand straight with your shoulders behind the line when the ball is thrown.
- Step, if necessary, on or over the line or to the side to catch the ball.
- Throw the ball hard enough so that it properly bounces back.
- Do not throw the ball too high for better rebounding.
- Catch the ball before it bounces on the ground.
- Catch the ball only with the hand, do not let the ball bounce against the body or your clothing.
- When the ball is thrown out of the deposition, the player may grab a new ball. These new balls are located in a box, on the inside of the fences. If all (six) balls are thrown out the fences, the attempt must be performed again.
- Encouragements and corrections are only allowed during the practice phase, NOT during the test attempt.

Practice phase:
The practice phase is first performed by all participating players. Every player should prior to the attempt throw and catch three times with both the left and the right. The player can choose to throw underhand or overhand him / herself. Interrupt the practice phase immediately if the player is standing over the line while throwing, letting the ball bounce against the body during catching, throwing too high, not getting to the ball when catching, not throwing hard enough and / or letting the ball bounce on the floor before catching. Then repeat the statement or a new demonstration. If necessary, provide for a practice attempt. After all the players have practiced, the test will be conducted.

Procedural errors:
When the player or the assessor make a mistake during the execution of the test or if there is a disturbing factor in the environment, we speak of a procedural error. When a procedural error happens the attempt must be terminated immediately and will not count in the score. The player may perform the test attempt again. However, when the process error is made because of 'incapability', the attempt is considered normal completed by the player. Examples of this are:

- The ball bounces for the catch;
- The player catches the ball through the clamp against the body or clothing;
- The player steps over the line while throwing;
- The player throws the ball alongside the table.

As an assessor, always be alert for possible misunderstanding of the player.

Score:
The number of correctly caught balls is noted within 30 seconds on the score sheet for both attempts.

Registration:
The number is correctly caught balls within 30 seconds of the two attempts recorded on the score sheet. The best attempt counts as final score.

Made by the Netherlands Table Tennis Association – Irene Faber The Netherlands, November 2012

Can Perceptuo-motor Skills Assessment Outcomes in Young Table Tennis Players (7-11 years) predict Future Competition Participation and Performance?: An Observational Prospective Study – PLOS ONE
Irene R. Faber*, Marije T. Elferink-Gemser, Niels R. Faber, Frits G.J. Oosterveld, Maria W.G. Nijhuis-Van der Sanden
*Faculty of Physical Activity and Health, Saxion University of Applied Sciences, Enschede, The Netherlands. E-mail: i.r.faber@saxion.nl.
