# Supplementary material for: Can Perceptuo-Motor Skills Assessment Outcomes in Young Table Tennis Players (7–11 years) Predict Future Competition Participation and Performance? An Observational Prospective Study
Source: PLoS One. 2016 Feb 10;11(2):e0149037. doi: 10.1371/journal.pone.0149037 (PMC4749309; doi:10.1371/journal.pone.0149037)
Supplement: S2 File — (PDF) [file pone.0149037.s002.pdf]

Research ID	Year of testing	Competition yes / no	Competition periods (n)	Training experience (months)	Current training (hours/week)	Sex	Test age (years)	Date of testing (dd.mm.yyyy)	Height (cm)	Weight (kg)	BMI (kg/m2)
1	2011	1	5	24	3	boy	9	27.03.2011	138	31,3	16,4
2	2011	1	5	36	5,5	boy	10	27.03.2011	150	36,3	16,1
3	2011	1	5	35	2,5	girl	10	27.03.2011	145	40,5	19,3
4	2011	1	5	12	1	boy	10	27.03.2011	144	34,7	16,7
5	2011	1	5	2	3	boy	9	27.03.2011	145	37,5	17,8
6	2011	1	5	12	2	boy	10	27.03.2011	161	55,5	21,4
7	2011	1	5	24	3	girl	10	27.03.2011	144	33,2	16
8	2011	1	5	6	7	boy	9	27.03.2011	144	34,3	16,5
9	2011	1	5	36	6	girl	9	27.03.2011	151	37,1	16,3
10	2011	1	1	18	2	boy	9	27.03.2011	134	31,7	17,7
11	2011	1	5	6	2	boy	10	27.03.2011	151	39,3	17,2
12	2011	1	5	16	1	girl	9	27.03.2011	148	39,9	18,2
13	2011	1	1	2	3	boy	10	27.03.2011	152	36,2	15,7
14	2011	1	5	12	2,5	girl	10	27.03.2011	143	42,9	21
15	2011	1	5	22	2,25	boy	10	27.03.2011	141	31,7	15,9
16	2011	1	4	16	1	girl	10	27.03.2011	151	48,2	21,1
17	2011	0	0	1	2,25	boy	11	27.03.2011	156	56,6	23,3
18	2011	0	0	6	2,5	girl	9	27.03.2011	147	38,3	17,7
19	2011	0	0	6	1	girl	9	27.03.2011	138	30,6	16,1
20	2011	1	5	12	1	girl	8	27.03.2011	137	33,2	17,7
21	2011	0	0	2	2,25	girl	9	27.03.2011	141	31,7	15,9
22	2011	1	4	2	2,25	girl	8	27.03.2011	134	34,6	19,3
23	2011	1	3	4	3	girl	8	27.03.2011	133	26,4	14,9
24	2011	1	4	12	1	girl	7	27.03.2011	126	29,5	18,6
25	2011	1	4	2	2,25	girl	7	27.03.2011	133	27,9	15,8
26	2012	1	5	9	3	boy	10	23.06.2012	155	45	18,7
27	2012	1	5	24	3	boy	10	23.06.2012	156	37,1	15,2
28	2012	1	5	12	3	girl	9	23.06.2012	138	27,4	14,4
30	2012	1	2	6	2,25	boy	11	23.06.2012	155	43,4	18,1
31	2012	1	5	13	3	girl	11	23.06.2012	158	49,6	19,9
33	2012	1	5	8	3	boy	10	23.06.2012	135	27	14,8
35	2012	1	5	8	3	girl	9	23.06.2012	144	32,6	15,7
36	2012	1	3	7	1	boy	10	23.06.2012	147	38,1	17,6
39	2012	1	2	7	1	boy	11	23.06.2012	144	32,2	15,5
40	2012	1	5	7	1	girl	10	23.06.2012	157	41,1	16,7
41	2012	1	5	7	1	boy	9	23.06.2012	145	34,2	16,3
42	2012	1	5	18	3	girl	11	23.06.2012	140	41,7	21,3
43	2012	1	5	18	3	boy	9	23.06.2012	135	31,1	17,1
44	2012	1	4	12	2	boy	9	23.06.2012	132	24,5	14,1
45	2012	1	1	6	3	boy	10	23.06.2012	149	31,2	14,1
46	2012	1	5	12	1,5	girl	9	23.06.2012	134	21,7	12,1
47	2012	0	0	3	2	boy	10	23.06.2012	153	32,8	14
48	2012	0	0	6	3	girl	9	23.06.2012	133	30	17
49	2012	0	0	3	1,5	girl	8	23.06.2012	129	26,4	15,9
50	2012	0	0	4	1	girl	9	23.06.2012	145	36,9	17,6
51	2012	0	0	2	1,5	boy	8	23.06.2012	140	31,8	16,2
52	2012	1	5	18	3	boy	7	23.06.2012	130	25,7	15,2
53	2012	1	1	3	1,5	girl	8	23.06.2012	131	29,6	17,3

1=yes
0=no

Can Perceptuo-motor Skills Assessment Outcomes in Young Table Tennis Players (7-11 years) predict Future Competition Participation and Performance?: An Observational Prospective Study – PLOS ONE
Irene R. Faber*, Marije T. Elferink-Gemser, Niels R. Faber, Frits G.J. Oosterveld, Maria W.G. Nijhuis-Van der Sanden
*Faculty of Physical Activity and Health, Saxion University of Applied Sciences, Enschede, The Netherlands. E-mail: i.r.faber@saxion.nl.

Research ID	Sprint (s)	Agility (s)	Vertical jump (cm)	Speed while dribbling (s)	Aiming at target (points)	Ball skills (points)	Throwing a ball (m)	Eye-hand coordination (catches)	Competitionscore				
									1	2	3	4	5
1	31	22	24	20	40	20	11	10	164	168	202	202	274
2	32	24	26	19	20	24	11	26	93	138	168	220	238
3	31	24	30	19	32	14	9	21	109	135	145	148	144
4	29	22	27	21	28	26	11,5	19	93	133	158	185	205
5	31	21	27	26	24	31	11	22	52	106	162	226	235
6	36	29	27	20	22	26	10	10	82	105	109	92	98
7	33	28	31	22	30	16	9,5	18	54	68	73	88	68
8	31	18	33	21	38	16	9,5	21	104	196	255	282	333
9	35	45	30	20	26	17	10,5	16	48	78	116	158	119
10	32	21	24	23	16	13	9	16	-28				
11	34	23	29	20	36	19	9	17	70	76	109	146	185
12	33	24	28	21	26	18	8	17	35	68	95	120	148
13	32	22	27	19	28	19	11	21	48				
14	35	21	35	36	20	18	9	3	20	36	48	73	105
15	34	26	17	31	10	21	7	10	-28	-40	52	38	63
16	37	29	28	28	18	17	8	13		39	49	68	83
17	32	23	29	31	12	29	11	18					
18	33	25	28	23	18	13	9	11					
19	37	35	19	23	12	12	8	6					
20	36	25	28	43	14	17	9	1	-12	-14	35	11	
21	31	21	28	25	24	11	9	12					
22	35	28	26	29	16	13	6,5	1	0	28	68	123	
23	38	29	27	33	12	22	8,5	8	-24	-20	-6		
24	44	28	20	39	16	10	7	3	-40	-40	-14	18	
25	31	23	24	32	30	17	7	7	-40	-5	68	137	
26	34	21	31	15	34	25	12	25	126	210	310	300	344
27	32	20	36,5	20	32	30	11,5	21	90	100	190	172	198
28	39	24	39	20	30	25	8,5	19	99	135	155	197	212
30	34	21	41,5	17	6	29	10,5	20				35	50
31	39	31	35,5	20	26	26	11	15	67	61	100	122	152
33	32	20	36	17	42	24	11	17	110	102	160	170	231
35	37	22	35,5	20	8	23	8,5	20	52	75	105	132	135
36	41	24	32	26	14	18	10,5	9	-14	18	23		
39	34	21	33	18	20	22	11	3	-26	18			
40	40	24	42,5	33	20	13	8,5	13	-38	12	8	-19	10
41	35	27	28,5	23	10	22	9	4	-46	16	0	1	18
42	38	30	32	22	4	10	8	14	-60	14	108	22	24
43	34	24	33	27	28	18	9,5	1	-54	1	-40	24	76
44	35	25	38	17	38	26	10,5	18		43	79	110	99
45	29	20	44	21	24	17	10	15			200		
46	36	25	35,5	25	34	15	9,5	17	10	69	160	206	283
47	37	26	29,5	22	8	22	9	12					
48	35	28	35	20	26	10	8,5	10					
49	37	27	33,5	37	23	5	7	2					
50	37	29	28	22	26	25	8	13					
51	33	28	30	24	18	23	10	11					
52	44	29	31	33	4	9	7	0	-80	-10	20	18	54
53	37	26	24,5	25	16	5	7	3				-13	

Irene R. Faber*, Marije T. Elferink-Gemser, Niels R. Faber, Frits G.J. Oosterveld, Maria W.G. Nijhuis-Van der Sanden
*Faculty of Physical Activity and Health, Saxion University of Applied Sciences, Enschede, The Netherlands. E-mail: i.r.faber@saxion.nl.
